# Pathophysiological Molecular Mechanisms of Obesity: A Link between MAFLD and NASH with Cardiovascular Diseases

**DOI:** 10.3390/ijms222111629

**Published:** 2021-10-27

**Authors:** Jorge Gutiérrez-Cuevas, Arturo Santos, Juan Armendariz-Borunda

**Affiliations:** 1 Department of Molecular Biology and Genomics, Institute for Molecular Biology in Medicine and Gene Therapy, University of Guadalajara, CUCS, Guadalajara 44340, Jalisco, Mexico; 2 Tecnologico de Monterrey, School of Medicine and Health Sciences, Campus Guadalajara, Zapopan 45201, Jalisco, Mexico; arturo.santos@itesm.mx

**Keywords:** obesity, comorbidities of obesity, metabolic dysfunction-associated fatty liver disease, nonalcoholic steatohepatitis, cardiovascular diseases, insulin resistance, pharmacological strategies

## Abstract

Obesity is now a worldwide epidemic ensuing an increase in comorbidities’ prevalence, such as insulin resistance, type 2 diabetes (T2D), metabolic dysfunction-associated fatty liver disease (MAFLD), nonalcoholic steatohepatitis (NASH), hypertension, cardiovascular disease (CVD), autoimmune diseases, and some cancers, CVD being one of the main causes of death in the world. Several studies provide evidence for an association between MAFLD and atherosclerosis and cardio-metabolic disorders, including CVDs such as coronary heart disease and stroke. Therefore, the combination of MAFLD/NASH is associated with vascular risk and CVD progression, but the underlying mechanisms linking MAFLD/NASH and CVD are still under investigation. Several underlying mechanisms may probably be involved, including hepatic/systemic insulin resistance, atherogenic dyslipidemia, hypertension, as well as pro-atherogenic, pro-coagulant, and pro-inflammatory mediators released from the steatotic/inflamed liver. MAFLD is strongly associated with insulin resistance, which is involved in its pathogenesis and progression to NASH. Insulin resistance is a major cardiovascular risk factor in subjects without diabetes. However, T2D has been considered the most common link between MAFLD/NASH and CVD. This review summarizes the evidence linking obesity with MAFLD, NASH, and CVD, considering the pathophysiological molecular mechanisms involved in these diseases. We also discuss the association of MAFLD and NASH with the development and progression of CVD, including structural and functional cardiac alterations, and pharmacological strategies to treat MAFLD/NASH and cardiovascular prevention.

## 1. Introduction

Obesity is a rapidly growing public health concern, and its worldwide prevalence has increased considerably in the last four decades, from less than 3% and 6% in 1975, to 11% and 15% in 2016, among males and females, respectively [1]. The pathophysiology of obesity involves the interaction of several factors such as environmental, socioeconomic, genetic, and internal, including alterations in central nervous system (CNS)-endocrine signals, intake of energy-dense foods, and decreased physical activity [2,3]. Therefore, a prolonged imbalance of energy intake and energy expenditure alters the metabolism and functions of white adipose tissue (WAT) in visceral fat depots, causing inflammation by an alteration in adipokines’ production, which are implicated in the development of many chronic metabolic diseases such as metabolic syndrome, type 2 diabetes (T2D), metabolic dysfunction-associated fatty liver disease (MAFLD), formerly named nonalcoholic fatty liver disease (NAFLD), nonalcoholic steatohepatitis (NASH), certain cancers, and cardiovascular disease (CVD) [3,4,5]. Moreover, specific adipokines secreted from adipocytes enhance the endothelial vasomotor tone by activating the renal renin-angiotensin system, which increases hypertension in obese patients [6]. Other comorbidities associated with obesity include sleep disturbance, respiratory difficulties, joint and mobility issues, psychological distress, and dyslipidemia [3].

The liver is the main organ for glucose and lipid metabolism, and it is often affected by obesity. When the hepatic fatty acid synthesis and/or uptake exceed the liver oxidative and/or its exportation capacity, lipid droplets are accumulated within the liver parenchyma, which cause MAFLD [7]. The association between MAFLD and obesity is evident because of bariatric surgery studies showed that MAFLD was detected in 85–95% of individuals with severe obesity. In addition, a meta-analysis study reported that 51% of MAFLD patients and 82% of patients with NASH were found to be obese [8]. MAFLD global prevalence is constantly increasing in parallel with the global obesity pandemic, and it is estimated to be approximately 25%, with the highest rates reported in South America and the United States (US), and the lowest in Africa, while the global prevalence of NASH ranges from around 3 to 5% [9,10]. Importantly, both metabolic syndrome and MAFLD independently increase the risk of T2D and CVD [11]. MAFLD is more prevalent in patients with major cardiovascular risk factors such as T2D, obesity, and hypertension [12]. MAFLD is associated to both peripheral and hepatic insulin resistance [13], and especially its advanced clinical manifestation of NASH exacerbates hepatic and peripheral insulin resistance, inducing atherogenic dyslipidemia and releasing pro-inflammatory factors, vasoactive factors, and thrombogenic molecules, which are involved with hypertension and the development of CVD, including coronary artery disease, and structural and functional cardiac alterations [14,15]. In fact, MAFLD subjects have an elevated risk of cardiomyopathies such as left ventricular (LV) systolic and/or diastolic contractile dysfunction and hypertrophy, which may lead to heart failure (HF) over time, as well as cardiac valvular calcifications (mainly aortic-valve sclerosis), arrhythmias such as atrial fibrillation (AF), corrected QT interval (QTc) prolongation, and ventricular arrhythmias, and certain types of cardiac conduction defects [16,17,18]. All of these additional MAFLD-related heart diseases could contribute to the high risk of CVD morbidity and mortality (approximately 40–45% of the total deaths) observed among individuals with MAFLD [16].

Progressive adipose tissue dysfunction and insulin resistance are key processes in NASH development and in hepatic fibrosis progression, supporting the existence of an adipose-tissue-liver crosstalk [19,20]. Several studies have associated NASH with atherosclerosis, increased coronary artery score, arterial stiffening, endothelial dysfunction, and myocardial dysfunction. This association is due to the increasing prevalence of NASH, caused by the global epidemic of obesity and diabetes [8].

CVD remains the leading cause of death in the world, and according to World Health Organization (WHO) statistics, 17.9 million deaths were caused by CVD in 2016, representing 31% of all deaths worldwide [21]. Obesity is an important risk factor for CVD, and atherosclerosis, hypertension, myocardial infarction, and cerebrovascular disease (stroke) are the most common CVDs. In addition, hypertriglyceridemia increases the incidence of CVD by 32% in men and 76% in women [22]. Additionally, cardiac fibrosis is common in CVD, including hypertension, myocardial infarction, and HF [21]. HF causes the most deaths globally and is commonly related with cardiac hypertrophy and cardiomyocyte apoptosis [23]. Therefore, CVD prevention and early detection and management of established cardiovascular risk factors, including insulin resistance, dyslipidemia, T2D, and hypertension, are crucial in public health policy.

In this review, we analyze the underlying pathophysiological mechanisms of obesity and its link with MAFLD, NASH, and CVD, and briefly summarize current pharmacological strategies to treat MAFLD/NASH and aid in cardiovascular prevention.

## 2. Obesity

### 2.1. Determination of Obesity

The first reports related to obesity were published at the end of the nineteenth century and the beginning of twentieth century [24,25,26]. In 1905, obesity was described by William Osler as morally reprehensible and medically undesirable [27]. Obesity results from energy excess (high sugar or fat intake), which is transformed to triglycerides and stored in adipose tissue, increasing body weight [28]. Therefore, obesity is defined by the WHO as an excessive fat mass that negatively affects health and is diagnosed by the determination of a body mass index (BMI) ≥30 kg/m^2^ (https://www.who.int/news-room/fact-sheets/detail/obesity-and-overweight, accessed on 28 August 2021). According to the WHO, the BMI is an imprecise measure of obesity because it does not take into consideration the body composition compartments; for instance, subjects with a similar BMI may have different degrees of fatness, and perhaps different metabolic profiles (https://www.who.int/news-room/fact-sheets/detail/obesity-and-overweight, accessed on 28 August 2021) [29,30]. Other studies have suggested measuring the waist circumference and waist-to-hip ratio to evaluate the visceral fat mass amounts better, and both are better predictors of mortality and morbidity than the BMI; furthermore, waist circumference is used in clinical and research settings to discriminate between overweight and obesity (https://www.who.int/publications/i/item/9789241501491, accessed on 28 August 2021) [31,32,33].

### 2.2. Etiology of Obesity

It is well-known that obesity is a multifactorial and largely preventable disease that results from complex relationships between several obesogenic factors, which are considered to influence the prevalence of obesity (Table 1). Furthermore, these factors might modify the epigenetic mechanisms to alter gene expression and thereby contribute more rapidly to the obesity phenotype [3,28,34,35,36].

### 2.3. Pathophysiology of Obesity

The pathophysiology of obesity involves a complex interplay of obesogenic factors, including those alterations in CNS-endocrine signals. The CNS detects information related to metabolic needs of adipose tissue, liver, stomach, muscles, and bones. In satiety conditions, hormones such as cholecystokinin, glucagon-like peptide-1 (GLP-1), insulin, and leptin are released to decrease food intake. Insulin and leptin are secreted primarily in response to glucose and the amount of adipose tissue, respectively. In contrast, the ghrelin, which is a potent orexigen, promotes food intake [2]. The basomedial hypothalamus detects deficiencies in nutrient supply; in this case, different groups of neurons such as agouti-related peptide/neuropeptide Y (AGRP/NPY) and pro-opiomelanocortin/cocaine and amphetamine regulated transcript (POMC/CART) are sensitive to leptin, ghrelin, insulin, and glucose, which are molecules signaling the availability of energy [37]. On the other hand, the sympathetic nervous system (SNS) is also involved in the restoration of energy balance, and its altered activity contributes to obesity. For instance, fasting decreases SNS activity, whereas the food intake, specifically carbohydrate overfeeding, increases SNS activity. Probably, the leptin and insulin mediate these effects on SNS activity [38]. Therefore, there exists an interaction between neurohormonal activation and obesogenic factors, which leads to further variance in phenotypic expression related with obesity.

The positive energy balance increases the volume of skeletal muscle, liver, among other organs and tissues of the body [39]. With respect to this, an obese individual with stable weight, compared with an individual with normal BMI, hence has greater fat and lean mass, which results in higher resting energy expenditure, cardiac output, and blood pressure, and greater pancreatic β-cell mass [39].

The de novo lipogenesis pathway synthesizes fatty acids from excess carbohydrates, and these fatty acids can then be incorporated into triglycerides for energy storage, and it has been hypothesized that the storage of triglycerides within adipocytes protects against lipotoxicity. In addition to being in the adipose tissue, these lipids are also found in liposomes (small cytoplasmic organelles), which are found in proximity to the mitochondria in many types of cells. Obesity can lead to an increase in the synthesis and secretion of low-density lipoprotein (LDL)-cholesterol and very-low-density lipoprotein (VLDL)-cholesterol, and low levels of the protective high-density lipoprotein (HDL)-cholesterol. VLDL particles can release the triglycerides by the endothelial lipoprotein lipase in extrahepatic tissues, of which adipose and muscle are primary tissues, and these tissues can use the triglycerides metabolically [39,40,41]. Moreover, the excessive triglycerides’ storage in obesity leads to the release of fatty acids by lipolysis, which is activated by the enhanced sympathetic state existing in obesity. High amounts of plasma free fatty acid (FFA) levels inhibit the lipogenesis, preventing appropriate clearance of serum triacylglycerol levels that conduce to hypertriglyceridemia and may result in insulin-receptor dysfunction [6].

Obesity causes profound microbial changes, and gut microbes impact the host metabolism affecting inflammation, fat deposition, and insulin resistance [41]. In addition, the microbiome composition and diversity are different between lean and obese subjects; however, these results have not been consistent [38]. In mice, fecal transplantation from an obese to a lean animal induces weight gain and obesity, while in humans, the transplanting of lean fecal microbiota into an obese individual improves insulin sensitivity and microbial diversity [38,41]. These findings suggest an important role for microbiota in the development of obesity.

Generally, obesity is associated with health problems; however, some individuals have a high BMI associated with a healthy metabolic profile and high insulin sensitivity, as well as a low amount of visceral adipose tissue (VAT), liver fat, and pro-inflammatory cytokine levels in plasma and adipose tissue [4]. Brochu and colleagues published the first original article on this topic in 2001 [42]. The definition of what is a healthy compared to an abnormal metabolic profile is much less consistent and controversial. For that reason, Ortega et al. proposed to harmonize the metabolically healthy but obese definition, considering it to be a man/woman with a BMI ≥30 kg/m^2^ and without any of the metabolic syndrome criteria, excluding waist circumference [43].

**Figure 1:**
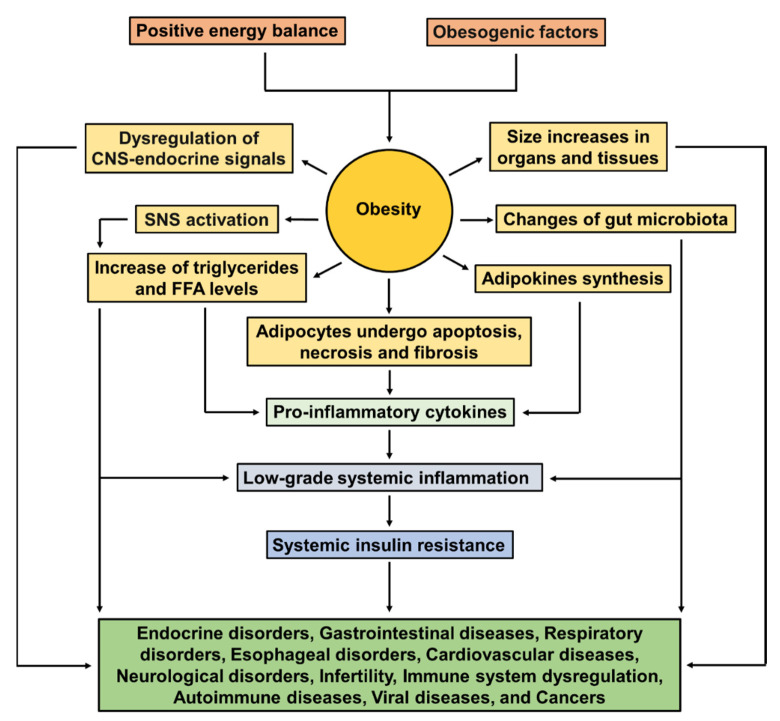
Pathophysiology mechanisms of obesity. Excess of energy-dense foods along with obesogenic factors induce obesity, which may cause several different disorders and diseases. Abbreviations: CNS, central nervous system; SNS, sympathetic nervous system; FFA, free fatty acid.

In persons with metabolically unhealthy obesity, excessive adiposity leads to adipocyte hyperplasia, which is mainly driven by the recruitment of adipogenic progenitors and growth factors, such as insulin-like growth factor-1 (IGF-1), tumor necrosis factor-α (TNF-α), angiotensin II (Ang II), and macrophage colony stimulating factor (M-CSF). The hypertrophied adipocytes undergo apoptosis, cell necrosis, and fibrosis, which further induce a low-grade systemic pro-inflammatory state due to an imbalance in adipokines’ production (adipocytes can secrete cytokines, hormones, and peptides); then the inflammatory response is intensified by macrophage recruitment to adipose tissue [4,44]. These macrophages and other immune cells produce pro-inflammatory cytokines such as leptin, resistin, IL-1b, IL-18, IL-6, and TNF-α, as well as inducible nitric oxide synthase (NOS), reactive oxygen species (ROS) and nitrogen intermediates (oxidative stress), and endothelial dysfunction. IL-6, IL-1b, TNF-α, and FFAs are responsible for activating c-Jun N-terminal kinase (JNK) and inhibitor κB kinase β (IKK-β). Activated JNK induces Ser 307 phosphorylation of insulin receptor substrate-1 (IRS-1), which alters insulin downstream signaling, and subsequently causes systemic insulin resistance, metabolic syndrome, and T2D [4,44,45]. Figure 1 illustrates common pathways by which the metabolic and physiological effects of excessive adiposity conduct to coexisting disorders and diseases in obesity.

### 2.4. Comorbidities Associated with Obesity

Obesity induces the neuro-hormonal activation and renin-angiotensin-aldosterone system (RAAS) activation, including sympathetic system activation, which along with hyperleptinemia and other homeostatic aberrations contribute to sodium retention and hypertension development; the latter with T2D and other conditions may lead to impaired renal function [41,44]. Obesity increases the risk of kidney stones and urinary incontinence in women [41]. In addition, the prevalence of obesity-related glomerulopathy increased in parallel with the global obesity epidemic [46]. It was also reported that being overweight or obese in midlife increases the risk of Alzheimer’s disease, vascular dementia, or any type of dementia [47]. Obesity is also associated with an increased prevalence of mood, anxiety, and other psychiatric disorders [34,39].

Obesity in women may cause preeclampsia and eclampsia in pregnancy, as well as a predisposition to depression, amenorrhea, menorrhagia, and infertility [6,41]. In men, obesity is associated with diminished sperm count and elevated rates of erectile dysfunction [41]. Excessive weight has been associated with hypothyroidism, Cushing’s syndrome, and polycystic ovary syndrome [34]. Obese patients with mild hypercortisolism, hypertension, and T2D may have Cushing’s syndrome [34]. Obesity increases blood volume, stroke volume, and cardiac output, which results in an increase in cardiac work. These alterations also lead to LV dilatation and left ventricular hypertrophy (LVH), which can favor the development of coronary heart disease (CHD) and other forms of CVD such as AF, HF, and ischemic stroke. Furthermore, an excessive body weight causes LV remodeling leading to both systolic and, particularly diastolic dysfunctions. In addition, obesity leads to enlargement of the left atrium, from both increased blood volume and diastolic ventricular dysfunction, which increases the risk of HF [4,41,48,49]. It is important to indicate that heart diseases, stroke, and chronic kidney diseases are all closely related to the pathophysiological mechanisms of high blood pressure.

Other alterations induced by obesity include an increase in pharyngeal soft tissues, which may block airways during sleep and cause obstructive sleep apnea that is associated with hypertension, systemic insulin resistance, liver dysfunction, inflammation, and dyslipidemia. Additionally, obesity also increases the risk of asthma, but the underlying mechanism is incompletely understood [39,41]. Obesity is also associated with a greater risk of pancreatitis and gallbladder disease [41]. An increased intra-abdominal pressure in persons who are overweight or obese is associated with elevated risks of gastroesophageal reflux disease, Barrett’s esophagus, and esophageal adenocarcinoma [39]. Interestingly, obesity accounts for approximately 20% of all cancer cases, such as esophageal, colon, pancreatic, endometrium, and renal cancers. Higher rates of overweight or obesity were also associated with gastric, uterine, gallbladder, cervical, and thyroid cancers, as well as advanced cancer of the prostate, including leukemia. Moreover, positive associations were seen with liver, ovarian (epithelial), and postmenopausal breast cancers [28,34,41].

Immune cells are abundant in adipose tissue, and the inflammatory response induced by obesity causes a low-grade systemic inflammatory state and a dysregulated immune system, which can be seen from childhood. This explains the increased risk of cancer and several kinds of infections, including surgical-site, urinary tract, nosocomial, and skin [41]. Obesity increases the risk of autoimmune diseases such as rheumatoid arthritis and osteoarthritis, which are associated to mechanical overload on joints. There is also strong evidence that obesity increases the risk of multiple sclerosis, psoriasis, and psoriatic arthritis [34,41].

Obese patients had a high risk of death during the H1N1 influenza pandemic in 2009 [50]. For the worst pandemic of humanity induced by the coronavirus disease 2019 (COVID-19), which is caused by the severe acute respiratory syndrome coronavirus 2 (SARS-CoV-2), it was reported that disease severity increased with BMI, and obese patients had worse outcomes with the COVID-19 infection, including respiratory failure, need for mechanical ventilation, and higher mortality [51,52,53]. In a Mexican population, the risk factors for lethality of COVID-19 patients were obesity, early-onset diabetes, hypertension, and chronic kidney disease, among others [54], obesity being the comorbidity more strongly associated with COVID-19 in Mexico [55].

MAFLD is associated with hypertension, dyslipidemia, T2D, and obesity. Around 20% of MAFLD cases progress to NASH [56]. In summary, the main overweight- and obesity-related comorbidities are described in Table 2.

## 3. Features of MAFLD and NASH

MAFLD is a common chronic liver disease in Western populations, and its severe phenotype NASH can potentially progress to fibrosis, cirrhosis, and hepatocellular carcinoma (HCC) [56]. Histologically, MAFLD occurs when ≥5% of the liver cells contain fat, and is severe when ≥30% of liver cells contain fat on a hepatic biopsy [57].

The prevalence of MAFLD is between 70% and 80% (even greater) among obese individuals or obese with T2D [16]. In 1979, Adler and Schaffner [58] published the association between obesity and MAFLD. Since then, MAFLD and NASH are often associated with the metabolic syndrome, which is characterized by central obesity, hypertriglyceridemia, low HDL-cholesterol, hypertension, and hyperglycemia [45,59]. Ludwing et al. [60] described a term of NASH in 1980. NASH is characterized by hepatic fat accumulation (macrovesicular steatosis), oxidative stress, hepatocyte injury (ballooning), lobular inflammation (usually in acinar zone 3, and the inflammation is followed by infiltration), and portal inflammation (usually mild), with or without fibrosis. Other histological lesions present in NASH include apoptotic bodies, sinusoidal collagen formation, Mallory–Denk bodies, megamitochondria, glycogenated nuclei, and iron deposition. While MAFLD is marked by macrovesicular steatosis without ballooned hepatocytes [59,61,62]. MAFLD/NASH is the third most common indication for liver transplantation in the US, and the second indication for liver transplantation in patients with HCC [63].

### Pathogenesis of MAFLD and NASH

**Figure 2:**
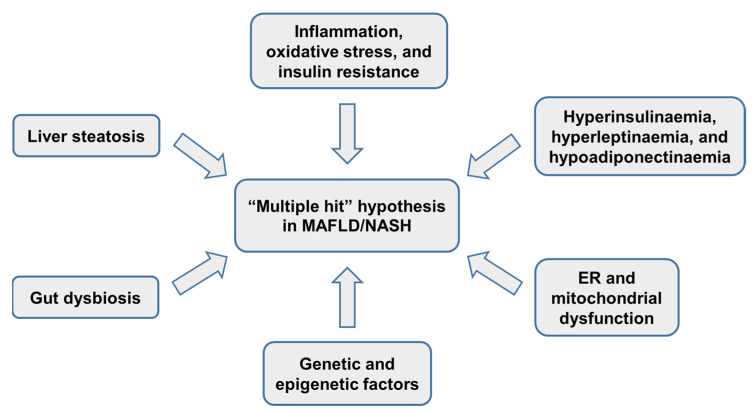
Multiples hits induce MAFLD and NASH. Lipids’ accumulation in the liver alters many different aspects of hepatocytes. Inflammation, oxidative stress, insulin resistance, hormones dysregulation, gut dysbiosis, organelle integrity and function, as well as genetic and epigenetic factors, are implicated in the development and progression of MAFLD and NASH. Abbreviations: MAFLD, metabolic dysfunction-associated fatty liver disease; NASH, nonalcoholic steatohepatitis; ER, endoplasmic reticulum.

The pathophysiology of MAFLD and NASH is complex and multifactorial. In 1998, Day et al. [64] proposed the “two-hit” hypothesis to explain the pathogenesis of MAFLD/NASH. The first hit leads to fat accumulation in hepatocytes, and the second hit causes inflammation and oxidative stress, which may cause fibrosis within the liver. Nowadays, a “multiple hit” hypothesis has been proposed to the pathogenesis of MAFLD/NASH; these hits include insulin resistance, among others (Figure 2) [64,65,66,67]. MAFLD appears when the rate of the hepatic synthesis of triglycerides exceeds non-esterified fatty acids’ catabolism, which depends on mitochondrial oxidation and their export as triglycerides in VLDL [68]. Excess fatty acids’ accumulation in the liver can occur through three pathways: lipolysis from adipose tissue, de novo lipogenesis, and the excess intake of dietary fat and sugars [8]. In NASH, the following alterations related to lipid metabolism are reported: (1) a decreased expression of acetyl-coenzyme-A carboxylase 1 (ACC1); ACC1 is the first key enzyme in de novo lipogenesis, and transforms acetyl-coenzyme-A into malonyl-CoA; (2) malonyl-CoA accumulation inhibits carnitine palmitoyltransferase (CPT)-1, which transports fatty acids into mitochondria, thus decreasing β-oxidation; and (3) the enzyme fatty acid synthase (FAS), which converts malonyl-CoA into palmitic acid, was found upregulated in simple steatosis individuals, and in NASH patients, it showed a deteriorated function [69]. In summary, the enzymes’ alterations avoid triglyceride synthesis, and this avoidance leads to the accumulation of toxic FFAs. NASH also includes an increase in hepatic free cholesterol, which in experimental models promotes hepatic inflammation and fibrosis [69,70]. In line with this, both sterol regulatory element-binding protein 2 (SREBP-2) and 3-hydroxy-3-methyl-glutaryl-coenzyme A (HMG-CoA) are increased in NASH patients; sterol regulatory element-binding protein 2 (SREBP-2) is a transcriptional regulator of 3-hydroxy-3-methyl-glutaryl-coenzyme A (HMG-CoA), which is a rate-limiting enzyme in de novo cholesterol synthesis [69,70]. Therefore, NASH is characterized by high triglycerides, low HDL-cholesterol, and increased LDL-cholesterol [71].

As was mentioned in the pathophysiology of obesity, pro-inflammatory mediators, such as leptin, resistin, IL-6, and TNF-α, may cause an adipose tissue dysregulation and a systemic insulin-resistant state [4,44]. Leptin has pro-fibrogenic effects in the liver of mice [8]. In patients with MAFLD, there is often an increase in IL-6, high sensitivity C-reactive protein (hs-CRP), IL-1b, TNF-α, chemokine (C-C motif) ligand 3, soluble intracellular adhesion molecule 1, and the macrophage phenotype 1/2 ratio (M1/M2) [17]. Adiponectin, which is an anti-inflammatory and anti-lipogenic adipokine, inhibits gluconeogenesis and causes FFAs oxidation, and it is decreased in NASH [45,72].

In NASH, there is also an increase in pro-inflammatory markers, such as C-reactive protein (CRP), IL-6, TNF-α, as well as pro-coagulant factors (fibrinogen, factor VIII, and plasminogen activator inhibitor-1), pro-oxidant molecules (oxidized LDL-cholesterol, thiobarbituric acid-reacting substances, and nitrotyrosine), and pro-fibrogenic mediators such as transforming growth factor beta-1 (TGF-β1), IGF-1, and endothelin-1 [71]. Fibrosis is a strong predictor of advancing NASH, and IL-6 concentration in serum and in liver tissue correlates with inflammation and fibrosis; meanwhile, ACC and FAS expression in NASH patients correlated inversely with the fibrosis stage [69]. TGF-β1 is the main stimulator of hepatic fibrosis, which is a pro-inflammatory and pro-fibrogenic pleiotropic cytokine that activates myofibroblastic hepatic stellate cells (HSCs) to secrete extracellular matrix proteins, mainly collagen, thus leading to fibrosis within the liver [70,73]. α-SMA is a marker of HSCs’ activation and can be used to assess the extent of liver fibrosis [74]. HSCs are generally quiescent in the sinusoids adjacent to hepatocytes, and when they are activated by signals of chronic liver injury, these cells are transformed to a myofibroblast-like phenotype and migrate to sites of tissue damage to express cytokines and chemokines that perpetuate inflammation and trigger fibrogenesis [73,75]. HSCs, monocytes, and Kupffer cells are the principal producers of TGF-β1; thus, a positive feedback loop exists between inflammatory and fibrogenic cells to stimulate each other in worsening fibrosis [73,75]. Interestingly, the Kupffer cells’ depletion increases insulin sensitivity and attenuates inflammation and fibrosis [76]. In adults with NASH, the fibrosis progression is characterized by the development of portal fibrosis, which may progress to cirrhosis. The absence of portal fibrosis in patients with NASH is indicative that patients’ development has fewer hepatic complications. In addition, fibrosis in adults with NASH is commonly pericellular and forms a dense, reticular network of “chicken wire” fibrosis, and it affects the lobular liver parenchyma. However, pediatric NASH is different with pure portal fibrosis [73]. It is important to note that the presence of advanced fibrosis is a significant predictor associated to an increase in cardiovascular death [77].

Necroptosis is implicated in chronic hepatic cell death and is proposed in the pathogenesis of NASH. The necroptosis pathway is regulated by distinct proteins such as receptor-interacting protein kinases 1 and 3 (RIPK1 and RIPK3) and downstream substrate pseudokinase mixed-lineage kinase domain-like (MLKL) [78]. In mouse models of NASH, such as in high-fat, choline-deficient diet-fed or methionine- and choline-deficient (MCD) diet-fed mice, RIPK3, MLKL, and TNF-α expression were found increased in the liver. RIP3K-dependent JNK activation promotes the release of pro-inflammatory mediators, which further maintain RIP3K-dependent signaling, cell death, and liver fibrosis. RIPK3 deficiency ameliorated MCD diet-induced liver damage, steatosis, oxidative stress, inflammation, and fibrosis [70,78].

Hypoxia impairs blood flow or obesity-associated obstructive sleep apnea [79]. Hypoxia also affects insulin signaling, and promotes the secretion of adipokines and inflammatory cytokines in adipose tissue, as well as inflammatory cytokines and pro-fibrogenic genes in the liver [70]. Moreover, nocturnal hypoxia triggers localized hepatic oxidative stress, and promotes the progression of pediatric nonalcoholic fatty liver disease [80].

Oxidative stress has a strong impact in the pathogenesis of NASH, which is generated by the excess FFAs’ load resulting from obesity and insulin resistance. ROS promotes to the lipid peroxidation of polyunsaturated fatty acids, and it produces toxic aldehyde products, causing damage to organelles, cell death, and the activation of fibrogenic HSCs [62]. Moreover, oxidative stress is linked with the induction of hepatocyte senescence, and several senescence-related proteins are associated with the progression of NASH disease; however, senescence of HSCs is considered to be necessary for the reversion of hepatic fibrosis and thus NASH resolution [73,81]. Obesity also induces endoplasmic reticulum (ER) stress, which is implicated in NASH. ER dysfunction, ATP depletion, including other stimuli, induce the unfolded protein response (UPR), which avoids the luminal accumulation of defective proteins and can induce apoptosis initiation. ER stress also induces other pathways that involve JNK, an activator of inflammation and apoptosis, and it is also implicated in the impairment of insulin signaling and development of diabetes and MAFLD progression to NASH. In addition, JNK activates SREBP-1c, which increases liver fat deposition, thus worsening ER stress [69,70,73]. Impaired autophagy is associated with the progression from steatosis to NASH; with respect to this, primary human hepatocytes exposed and treated with palmitic, oleic, and elaidic acid showed a diminution in lipid content and ER stress with an induction of autophagy when treated to GLP-1 agonist exendin-4 [69].

The gut microbiota has been involved in NASH progression because intestinal dysbiosis can trigger intestinal inflammation and impair the gut barrier, affecting the digestion and absorption of nutrients, the host immune system, and the secretion of gut hormones. Microbial products may reach the liver, induce hepatic inflammation, and contribute to MAFLD and NASH progression. The intestinal microbiota in MAFLD with advanced liver fibrosis was accompanied by pro-inflammatory bacteria such as Escherichia coli and Proteobacteria [16]. Bacteroides were highly abundant in NASH, whereas Prevotella was low in NASH patients. Furthermore, western diets rich in fat, animal proteins, and sugar favor Bacteroides in NASH [82].

## 4. Obesity and Cardiovascular Diseases

Obesity is an independent risk factor for CVD and, as noted above, increases the risk of diseases such as dyslipidemia, insulin resistance, hypertension, and atherosclerosis both in children and adults [83,84,85]. Several studies have shown an association between obesity and cardiovascular diseases such as stable coronary disease, acute myocardial infarction, HF, cardiac arrhythmias, and sudden cardiac death [86], which are the main causes of death among the diabetic population.

The complex interaction between adipose tissue and the cardiovascular system plays an essential role in the pathogenesis of CVD. In 1847, an autopsy of a severely obese man showed that the heart was large, thick, and fibrous, and was filled with fat. In 1933, other autopsies performed on obese individuals revealed that the heart weight was greater than that expected for normal weight, and most obese persons (95% of cases) had excessive epicardial fat, manly over the right ventricle. In 1955, it was reported that a severely obese man displayed HF, severe pulmonary hypertension, and high cardiac output (CO) [87]. In peripheral obesity, the high adipose deposition promotes to an increase in blood volume, which predisposes to an increase in CO. Furthermore, the increase in CO in peripheral obesity is due to the augmentation of LV stroke volume [88]. Subsequent autopsies confirmed increased heart weight and LV and right ventricular hypertrophy, which were related to the degree of obesity. Additionally, cardiac fibrosis was found in both animal models and clinical studies of obesity [89]. Furthermore, obesity-induced alterations in the myocardial structure include LV remodeling with increased wall thickness and mass, including LVH; moreover, right ventricular thickness and volume are also increased [87,89]. Left atrial enlargement (LAE) is associated with obesity in both children and adults, occurring commonly in normotensive obese subjects. The Framingham observational cohort study reported a strong association between LAE in obese individuals and incidence of AF [87,90].

Obesity is associated with HF events, and incidence of HF is higher in diabetic patients. The changes related to cardiac fibrosis and stiffness, as well as subclinical diastolic dysfunction, often progress to HF with preserved ejection fraction (HFpEF), including eventual systolic dysfunction accompanied by HF with decreased ejection fraction (HFrEF) [88,91]. Intriguingly, although obesity is considered an independent risk factor for the development of HF, it is commonly associated with improved prognosis in the setting of existing HF; this is referred to as the “obesity paradox” of HF [91]. LV diastolic dysfunction can occur independently or in conjunction with systolic dysfunction. It is common in obese persons, especially in extremely obese and in hypertensive obese individuals; probably the most important factor that contributes to LV diastolic function is LVH [87,89,90]. LV systolic function was found to be either normal or supranormal in obese individuals. Mildly decreased LV systolic function was showed in Zucker rats with obesity and diabetes, transgenic mice, and different mouse models with insulin resistance and myocardial steatosis associated to a chronic high-fat diet [90]. However, growing evidence has identified a subclinical depression of LV systolic function, particularly in morbidly obese subjects [89]. Other factors contributing to LV systolic dysfunction in obesity include adverse LV loading conditions, duration of obesity, and increased LV mass, including comorbidities such as coronary artery disease, hypertension, and diabetes [87].

### 4.1. Pathogenesis of Obesity in Cardiovascular Diseases

In conditions of obesity, insulin resistance, or diabetes, the heart tissue is exposed to high levels of fatty acids and carbohydrates, and lipids are deposited in vesicles in the myocardium (cardiac steatosis) due to an imbalance between the uptake and β-oxidation of fatty acids [92]. It was proposed that protein-mediated fatty acid uptake implicates the binding of the fatty acid to the fatty acid binding protein (FABPpm), followed by actual transport and uptake via the cluster of differentiation 36 (CD36, also named fatty acid translocase, FAT) or fatty acid transport proteins (FATP1/6) [93]. FAT/CD36 is also important in AMP-activated protein kinase (AMPK)-mediated stimulation of FFAs’ uptake in cardiomyocytes [88]. Saturated long chain FFAs, especially palmitic acid, which predominates in epicardial fat, are the main contributors to systemic lipotoxicity compared to long chain monounsaturated FFAs such as oleic acid [94]. Hyperlipidemia and hyperinsulinemia both stimulate FFA transport into cardiomyocytes, and an excess of lipids’ accumulation causes cardiac dysfunction via several mechanisms, including the generation of ROS and the production of lipid metabolites such as diacylglycerols, ceramides, or acylcarnitines. These metabolites further impair insulin signaling, as well as depress contractility by influencing sarcoplasmic reticular Ca^2+^ stores and promoting mitochondrial dysfunction, ER stress, or apoptosis [49,88,91,95]. In addition, ceramides and diacylglycerols activate different protein kinase C isoforms, such as PKCβ, PKCδ, or PKCθ. The PKCs’ activation promotes inflammation, fibrosis, and cell death, which each contribute to cardiac dysfunction [95]. Furthermore, diacylglycerol induces lipid-associated ER stress, activating PKCε, and inducing insulin resistance as well as lowering nitric oxide production. Meanwhile, ceramida may activate PKCs and inhibit insulin metabolic Akt/PKB signaling (Akt: phosphoinositide dependent kinase), downregulating glucose transporter 4 (GLUT4) translocation and insulin-stimulated glucose uptake in heart tissue with diabetes [88]. Obesity can cause alterations in insulin sensitivity, and insulin-resistance in the heart has important implications for cardiac metabolism and function. Hyperglycemia promotes the production of ROS, which may induce apoptosis and activate poly (ADP-ribose) polymerase-1 (PARP). Glyceraldehyde phosphate dehydrogenase (GAPDH) is ribosylated and inhibited by PARP; then glucose is deviated from the glycolytic pathway toward other biochemical pathways, causing hyperglycemia-induced cellular damage. Moreover, hyperglycemia also alters cardiac structure and function through post-translational modification of extra-cellular matrix proteins, and it impairs expression/function of intramyocellular calcium channels, promoting systolic and diastolic dysfunction [91].

The accumulation of lipids within cardiomyocytes as well as visceral adiposity are associated with the expansion of epicardial and pericardial fat. These lipid depots may provide fatty acid substrates to the myocardium, but they also play a role in the elaboration of pro-inflammatory cytokines and adipokines that impact coronary and myocardial function [49,96]. It is well-known that FFAs are important adipocyte-derived mediators of macrophage-related inflammation, and macrophages may be important mediators of saturated FFAs’ effects on cardiac electrical remodeling [94]. The NF-κB transcription factor is critical for the regulation of cardiac inflammatory signaling pathways and is involved in cardiac remodeling that leads to the pathogenesis of HF. Furthermore, NF-κB plays a role in the hypertrophy of primary rat neonatal ventricular cardiomyocytes in response to Ang II, phenylephrine, and endothelin-1 [94]. IL-6 is a pleiotropic cytokine stimulated by distinct saturated FFAs in macrophages via TLR4 activation, and it is an indicator of cardiovascular risk [95]. Additionally, in a multiethnic study of atherosclerosis, IL-6 expression was increased in obese patients and correlated strongly with incidence of HF [96]. The TNF-α signaling pathway has been studied in the cardiac dysfunction related with cardiac ion channel remodeling in animal models [94], but its role on cardiac function related to lipotoxicity is poorly understood. In the heart, myocardial inflammation and inflammatory cell infiltration, including the expression of pro-inflammatory cytokines such as TNF-α, IL-6 and IL-8, monocyte chemotactic protein 1 (MCP-1), intercellular adhesion molecule 1 (ICAM-1), and vascular cell adhesion molecule 1 (VCAM-1), contribute to cardiac oxidative stress, and promote ROS generation and alter calcium, as well as affect the downregulation of SERCA2 by IL-1β, thus impairing myocardial relaxation in the early stage of diabetic cardiomyopathy [88,92]. Under the establishment of fibrosis, the damaged myocardium secretes pro-fibrotic molecules such as Ang II, TGF-β1, and IL-1β to promote chronic inflammation, causing aberrant NLRP3 inflammasome activation [97].

Lipids’ accumulation within the myocardium also causes mitochondrial dysfunction, which is involved in diabetic cardiomyopathy and HF [88]. In response to mitochondrial dysfunction, the removal of damaged mitochondria is directed by mitophagy (autophagy of mitochondria). Mice fed with a high-fat diet (HFD) showed at 6 weeks a declination of cardiac autophagic flux. In contrast, it was reported that mitophagy began at 3 weeks after the start of the HFD and lasted well after 2 months, and this finding was accompanied by cardiac hypertrophy and diastolic dysfunction, including diabetic cardiomyopathy [98]. Lipotoxic conditions also generate lipid peroxidation products such as 4-hydroxy nonenal (4-HNE), 4-hydroxy hexenal, and oxidized cardiolipina that damage cellular membranes and alter organelle function. In line with this, a high fat/high sucrose diet induces cardiac hypertrophy and fibrosis, including increased 4-HNE adducts and protein carbonyls [96,99]. In summary, the inflammatory signaling pathways are involved in mitochondrial dysfunction, ER stress, remodeling and cardiac fibrosis, cell death, as well as diastolic and systolic dysfunction [49,88,92].

### 4.2. Altered Lipid and Lipoprotein Metabolism in the Liver Contributes to CVD Risk

Dyslipidemia is very common in MAFLD patients, which is characterized by hypertriglyceridemia, with moderately increased LDL-cholesterol and decreased HDL-cholesterol [100]. Meanwhile, elevated levels of LDL-3 and -4 particles (atherogenic forms of LDL) and reduced HDL2b levels (a cardioprotective lipoprotein) are founded in NASH [101,102]. With respect to this, dyslipidemia is closely linked to CVD, and LDL-cholesterol is the main driver of atherosclerosis [100]. In addition, MAFLD severity is positively correlated with high ratios of total cholesterol/HDL-cholesterol, LDL-cholesterol/HDL-cholesterol, triglycerides/HDL-cholesterol, and non-HDL-cholesterol/HDL-cholesterol, which is an atherogenic lipid profile [103]. One study found few differences in cholesterol profiles between the nonalcoholic fatty liver and NASH patients, indicating similar cardiovascular risk profiles [104]. The high levels of VLDL particles in MAFLD increase to small dense LDL (sdLDL) particles, which result in the characteristic atherogenic dyslipidemia pattern. These sdLDL particles and ApoB-containing particles are responsible for the formation of atherosclerotic plaque in the vascular endothelium and contribute to increased risk for CVD [100]. Even other studies indicate that plasma ApoB concentration is a stronger predictor of CVD risk than LDL-cholesterol or plasma triglycerides levels [105]. In addition, MAFLD rather than advanced hepatic fibrosis is independently associated with an elevated prevalence of chylomicrons plus VLDL remnants, VLDL, LDL, and VLDL plus LDL dyslipoproteinemias, suggesting that ApoB dyslipoproteinemias may contribute to increased CVD risk associated with MAFLD [106]. In line, one study assessed cardiovascular disease risk by lipoprotein profile in 76 children with MAFLD and reported that the severity of hepatocyte ballooning was associated with higher ApoB/ApoA1 ratios, a finding which suggests that the severity of the histologic features is closely associated with the severity of cardio-metabolic risk [107]. On the other hand, reverse cholesterol transport (RCT) is an important cardioprotective process, and obesity impairs RCT due to reduced plasma cholesterol uptake and efflux by hepatocytes and adipocytes. Therefore, a reduction in the capacity for plasma cholesterol clearance may partly account for the increased CVD risk with obesity [108]. In line, one function of HDL-cholesterol is the ability to promote RCT, and low HDL-cholesterol is a marker for MAFLD and a risk factor for CVD [109].

In summary, several findings of cross-sectional studies, meta-analyses, and systematic reviews suggest that MAFLD increases the risk of atherosclerosis and the development of unstable plaques. In support to this, genetic evidence suggests that MAFLD-caused dyslipidemia is a main factor of elevated CVD risk [109]. In addition, MAFLD progression to NASH and fibrosis is heterogeneous and occurs over years or even decades, and the evolution of MAFLD is related to alterations in hepatic and extra-hepatic lipid metabolism, and the altered glucose metabolism and insulin resistance (an important driver of MAFLD and NASH) exacerbate CVD risk in these patients [105]. Furthermore, other processes such as hyperglycemia, oxidative stress, inflammation, and endothelial dysfunction create a pro-atherogenic environment, favoring CVD development [105].

## 5. Association of MAFLD and NASH with Cardiovascular Diseases

The dysfunction of the VAT, including an increased accumulation of ectopic fat in the liver and other organs, such as the pericardium, pancreas, kidneys, or skeletal muscle, is closely associated to adverse cardio-metabolic outcomes [15]. In a longitudinal cohort, increasing hepatic fat over 6 years was associated with the progression of cardiovascular risk factors, even after accounting for BMI changes [110]. With respect to epicardial adipose tissue (EAT), it is the visceral fat depot of the heart and produces both pro-inflammatory and anti-inflammatory mediators [1,49]. One study with 868 subjects reported that hepatic steatosis and epicardial fat thickness were related to an increased incidence of extra-cardiac plaques [57]. Patients with MAFLD and thicker EAT (>3.18 mm) are at increased risk for coronary calcification [17]. One meta-analysis with 13 case-control studies (*n* = 2260 patients) reported that EAT was significantly increased in MAFLD patients compared with controls. Furthermore, EAT correlated with the severity of liver steatosis and fibrosis, and atherosclerotic CVD [1]. In line, a meta-analysis study, which included a total of 13 case-control studies (*n* = 2260 patients), found that EAT was associated with the severity of steatosis, fibrosis, and cardiovascular disease in patients with MAFLD [111].

Several mechanisms contribute in MAFLD to CVD risk, such as hepatic insulin resistance, systemic low-grade inflammation, adhesion molecules, and a pro-thrombotic state [71]. Furthermore, individuals with MAFLD show endothelial dysfunction with a significant decrease in brachial artery endothelial flow-mediated vasodilatation [112]. One Korean study included 334,280 healthy subjects, and the fatty liver index was used to identify subjects with MAFLD. Interestingly, the study showed that healthy MAFLD patients progressed to develop CVD independently of the interim occurrence of other metabolic diseases [113]. In addition, the prevalence of steatosis and of severe fibrosis in an asymptomatic general adult population was estimated, and cardiovascular risk (CVR) was evaluated by atherosclerotic cardiovascular disease. In this adult population, the presence of MAFLD and severe fibrosis was associated with a higher CVR profile [114]. Another retrospective cross-sectional study of biopsy-proven liver steatosis assessed CVR and CVD between 109 patients who met diagnostic criteria for NAFLD and 154 for MAFLD. Patients with both NAFLD and MAFLD showed intermediate/high CVR, with a high rate of CVD [115].

MAFLD is an independent risk factor for atherosclerosis and CVD and is often associated with the main risk factors for atherosclerosis and CVD, such as dyslipidemia, T2D, and hypertension [68]. However, it was reported in one study involving 102 patients with T2D that hepatic steatosis was not associated with an increased risk of CVD. In contrast, a cross-sectional study on 151 subjects showed a strong association between MAFLD and atherosclerosis independent of T2D [57]. A meta-analysis of 26 cross-sectional studies with approximately 85,000 individuals showed a close association between MAFLD and markers of subclinical atherosclerosis. These results showed mainly an association with greater carotid artery intima-media thickness/plaques, increased arterial stiffness, coronary artery calcification, and circulatory endothelial dysfunction. In addition, some observational studies, performed on Asian individuals, showed a strong association between the severity of MAFLD and the long-term risk of progression of subclinical coronary or carotid atherosclerosis. Moreover, the resolution or improvement in MAFLD confirmed by ultrasound examinations was associated with a lower risk of carotid atherosclerotic development [116]. In a nested cohort study from the US with 3756 individuals, who were evaluated by coronary contrast-enhanced computed tomography angiography, it was reported that MAFLD remained associated with a higher risk of incident CVD events after adjustment for established cardio-metabolic risk factors and the extent of coronary plaques and stenosis [117]. Recently, in a cohort of approximately 300 adults from the US with biopsy-confirmed MAFLD (without CVD), the severity of hepatic fibrosis was associated with an approximately 2.9-fold increased risk of incident CVD events over a median of 5.2 years [118]. Furthermore, the steatosis severity of MAFLD is independently associated with coronary artery atherosclerosis, but it is still controversial. In Taiwanese individuals (*n* = 1502), the presence of atherosclerotic plaques correlated with the severity of steatosis, especially high-risk plaques, and was independent of traditional risk factors [119]. In addition, CHD is associated with MAFLD in patients without hypertension and diabetes [120]. Another study, which included patients diagnosed with MAFLD in primary care between 2010 and 2015, reported that MAFLD constitutes an independent risk factor for CHD, myocardial infarction, and atrial fibrillation (AF) in Germany [121]. Since controversy still stands on whether or not NAFLD/MAFLD raises the odds of CVD, the prevalence of fatty liver disease and the associated CVD risk was evaluated using each of these definitions, respectively. NAFLD and MAFLD were each associated with significantly higher risk for CVD events [122]. In addition, the risk for adverse cardiovascular events (CVEs) in elderly acute myocardial infarction patients who also had MAFLD was significantly higher [123].

Although considerable studies have demonstrated increased cardiovascular mortality in MAFLD patients, the independent contribution of MAFLD to CVD mortality remains controversial [8,17,124,125]. A comprehensive meta-analysis of 16 longitudinal studies with around 34,000 individuals confirmed that imaging-defined or biopsy-proven MAFLD was associated with an approximately 1.6-fold increased risk of fatal and/or non-fatal CVD events over a median of 6.9 years (OR 1.64, 95% CI 1.26–2.13). Moreover, other prospective studies have confirmed that the risk of incident CVD events paralleled the underlying severity of MAFLD, and that the stage of liver fibrosis was a key prognostic marker for long-term CVD outcomes and overall mortality in individuals with MAFLD [116]. Recently, one study assessed the association of atherosclerotic cardiovascular disease (ASCVD) risk scores with overall and cardiac-specific mortality among patients with MAFLD. Among patients with MAFLD, ASCVD was associated with a higher risk of overall and cardiac-specific mortality [126]. Kim et al. [127] studied the impact of MAFLD and NAFLD on the all-cause and cause-specific mortality in US adults. MAFLD was associated with increased risk of all-cause mortality, while NAFLD demonstrated no association with all-cause mortality after adjusting for metabolic risk factors. Therefore, MAFLD may provide a better understanding of predictors that may increase the risk of death. Other studies have suggested that the increased risk and mortality of CVD may be limited to MAFLD patients with advanced fibrosis and T2D [15,77,124,128]. For instance, patients with liver fibrosis in the stage 3 or 4 had increased mortality regardless of the MAFLD activity score (HR 3.3, CI 2.27–4.76, *p* < 0.001) [124]. Interestingly, a couple retrospective studies with a long duration of follow-up demonstrated that individuals with NASH, but not those with simple steatosis, were at higher risk of CVD mortality [71]. Another prospective American study found that NASH increases liver-related mortality, but not overall mortality when compared with MAFLD. However, NASH was not found to be linked with an increase in CVEs after adjusting for BMI and T2D, but in this study, there was a low prevalence of CVEs overall [125].

NASH may contribute to systemic low-grade inflammation and to cardio-metabolic disease through the proteins secreted by the liver, such as IL-6, CRP, fibrinogen, MCP-1, TNF-α, β-trophin, and fetuin-A [68]. On the other hand, lipoprotein(a) (Lp(a)) is an important independent cardiovascular risk factor. However, advanced NASH is associated with low serum Lp(a) levels; therefore, Lp(a) levels may not be useful in evaluating cardiovascular risk [129]. The severity of liver fibrosis is used to evaluate prognoses for patients with MAFLD. It was shown that individuals with MAFLD had more than a 2-fold increase in the risk of CVEs, and those with liver fibrosis had a 4-fold increase in risk. Furthermore, patients with MAFLD and liver fibrosis indexes were independently associated with risk of incident CVEs [130].

Ultrasound imaging is used as non-invasive tool to detect hepatic steatosis, but its sensitivity is poor when hepatic steatosis is minor to 30% [131]. With respect to this, MAFLD was reported as a risk factor for atherosclerosis as defined by measurements of carotid intima-media thickness (CIMT) and coronary artery stenosis of greater than 50%. However, the studies used ultrasound imaging to establish MAFLD without considering the prevalence of NASH [125]. Other meta-analysis studies also found a significant association between MAFLD and increased CIMT, including an increased prevalence of carotid atherosclerotic plaques [14,57,125]. The severity of MAFLD has been strongly associated with a more atherogenic lipid profile and increased arterial stiffness. Moreover, increased hepatic stiffness was related with high coronary-artery calcium scores in MAFLD individuals, independently of cardio-metabolic risk factors [14]. Furthermore, it was reported that atherosclerosis and alterations of cardiac function as well as impaired flow-mediated vasodilation and increased CIMT may occur already in obese children and adolescents [132]. MAFLD was found associated with calcification in both the aortic and mitral valves in patients with and without T2D [16]. Some evidence supports the existence of a significant association between MAFLD and risk of aortic-valve sclerosis, which is associated with CVD morbidity and mortality [116]. Furthermore, aortic-valve sclerosis and mitral annular calcification are risk factors for cardiac arrhythmias [16]. In a study of middle-aged subjects in Germany, it was reported by ultrasonographic diagnosis that MAFLD was associated with an increased prevalence of aortic-valve sclerosis, independent of cardio-metabolic risk factors [16]. Several studies have found a relation between coronary artery calcification (CAC) and MAFLD; however, some studies did not find an association [17,133,134]. Recently, a cross-sectional analysis of well-characterized, prospective cohorts including 105 patients determined the association of high-risk CVD phenotype by CAC with liver fibrosis by magnetic resonance elastography (MRE) in patients with MAFLD. This study found that CAC was more prevalent in patients with significant fibrosis than those without as evaluated by MRE [135]. Therefore, patients with MAFLD and significant fibrosis by MRE should be considered for further cardiovascular risk assessment. In the Framingham Heart Study, among 3276 adult participants with MAFLD, the liver stiffness was assessed by vibration-controlled transient elastography, and hepatic fibrosis was associated with multiple cardiovascular risk factors, including obesity, metabolic syndrome, diabetes, hypertension, and HDL-cholesterol [136]. In line with this, one retrospective observational cohort study, which included 41,005 patients with NASH, of which 15,758 were male and 25,247 were female, was conducted. The study found that CVD risk factors and diseases were highly prevalent, hypertension being the most prevalent CVD risk factor (68%), followed by diabetes mellitus (62%), obesity (40%), dyslipidemia (37%), smoking (30%), and renal failure (27%). In addition, men had higher rates of most risk factors and diseases compared with women, except for higher rates of obesity and diabetes in women [137]. Furthermore, in adults with biopsy-proven MAFLD, advanced fibrosis on the biopsy and a higher MAFLD fibrosis score were significant and independent predictors of incident cardiovascular disease, even after considering traditional risk factors and cardiovascular risk scores [118]. Therefore, these findings should be considered when evaluating MAFLD patients for the primary prevention of cardiovascular disease.

### 5.1. Ischemic Stroke

Several studies have demonstrated high levels of aminotransferase and g-glutamyltransferase (gGT) in MAFLD patients, which are associated with increased incidence of ischemic stroke. Moreover, gGT has been isolated from atheromatic plaques, macrophages, and foam cells, and it appears to contribute to atherosclerosis through the induction of oxidative stress [12]. However, in data from a Korean prospective cohort study, the level of alanine transaminase (ALT), aspartate transaminase (AST), or gGT alone did not show any significant association with stroke in patients with MAFLD. Moreover, this study demonstrated that the risk of stroke incidence gradually increased with the degree of the fatty liver index [138]. Recently, in a community-based cohort study involving around 80,000 Chinese subjects followed for a median of 10.3 years, it was reported that the ultrasonographic severity of MAFLD was associated with a higher risk of future ischemic stroke events, independently of common CVD risk factors [139]. On the other hand, MAFLD was not associated with a higher incidence of stroke in a primary care population in Germany [121]. However, a meta-analysis of nine case-control and cohort studies reported that MAFLD was independently associated with 2.3-times higher risk for ischemic stroke (95% CI: 1.84–2.93), and this association was independent of traditional cardiovascular risk factors, including dyslipidemia, obesity, and T2D. In another case-control study, made up of 295 patients with acute ischemic stroke and 1942 subjects with a health check-up, the degree of liver fibrosis analyzed by transient elastography was independently associated with increased stroke risk [12]. In line with previous studies, the severity of MAFLD is associated with a higher risk of future ischemic stroke events, and MAFLD-fibrosis may be associated with stroke in addition to heart disease, with differences depending on the measure used to define MAFLD-fibrosis [140,141]. With respect to this, advanced liver fibrosis was not significantly associated with ischemic stroke overall using the Fibrosis-4 and nonalcoholic fatty liver disease fibrosis score. Furthermore, advanced liver fibrosis may be associated with a higher risk of ischemic stroke in women but not men [142].

### 5.2. Structural Cardiac Abnormalities

Several studies have shown in MAFLD patients a higher prevalence of LV wall thickness and myocardial mass [17]. In addition, a project of the Framingham Heart Study with 2356 participants showed that liver fat was positively associated with LV mass, LV wall thickness, mass volume ratio, mitral peak velocity (E), and LV filling pressure (E/e’ ratio) [143]. MAFLD also was found independently associated with a higher prevalence of LVH in hypertensive T2D individuals [124]. In addition, in a prospective study of 1827 black and white middle-aged adults followed for 5 years, it was reported that imaging-detected MAFLD was associated with an increased risk of incident LVH, abnormal LV geometry, and impaired LV function, independent of established risk factors for HF [144]. The MAFLD fibrosis score was correlated with a worse outcome in 492 patients with HFpEF [124]. In addition, patients with NASH were associated with cardiac abnormalities such as left atrium enlargement and increased LV mass [124].

### 5.3. Cardiac Arrhythmias

Several studies found a strong association between MAFLD and a higher risk of certain arrhythmias, such as permanent AF, QTc interval prolongation, and ventricular tachyarrhythmias [116]. For instance, the association of hepatic fat and LV filling pressure was only partially mediated by BMI, suggesting a possible direct effect of liver fat on LV filling pressure (E/e’ ratio) [143]. A reduced myocardial mechano-energetic efficiency (MEE) has been found to be an independent predictor of CVD. It was shown that the ultrasound-defined presence of MAFLD is associated with a decreased MEE [145]. On the other hand, pericardial fat is associated with the elevated prevalence of AF [146]. A study conducted by Mahfouz et al. on 260 individuals found that increased interatrial thickness and the left atrial stiffness index could be related with an increased incidence of AF in MAFLD patients [57]. Several studies have reported that MAFLD had an increased prevalence and correlation with an increased risk of AF, especially in T2D patients [14,57,124]. Recently, in a meta-analysis of nine observational studies (five cross-sectional and four longitudinal), it was reported that MAFLD was associated with an approximately 2-fold increased risk of prevalent AF (OR 2.07, 95% CI 1.38–3.10), independently of common risk factors for AF [116]. A project of the Framingham Heart Study with 3744 participants showed that 383 participants expressed high levels of ALT or AST, and they had an increased independent risk for developing AF at the 10-year follow-up [17]. In line, a retrospective study of 267 consecutive patients undergoing AF ablation showed that MAFLD is associated with significantly elevated arrhythmia recurrence rates following AF ablation during a mean follow-up of 2 years [147]. Moreover, a prospective multicenter cohort study, which included patients with nonvalvular AF who received vitamin K antagonists (VKAs) or non-VKA oral anticoagulants, showed that MAFLD was highly prevalent in AF but was not associated with higher bleeding or thrombotic risk [148]. Cai et al. [149] reported in a total of six cohort studies with 614,673 individuals that MAFLD is associated with increased risk of incident AF, and the strength of the association between MAFLD and AF is partially attributed to the co-existing cardio-metabolic risk factors. Furthermore, AF was found to be independently associated with advanced liver fibrosis in patients with MAFLD [150]. Therefore, MAFLD and NASH markedly increase the risk of atrial fibrillation.

Additionally, MAFLD individuals show an increased prevalence of premature ventricular beats (19.3% vs. 6.5% in patients without MAFLD, *p* < 0.005), as well as non-sustained ventricular tachycardia (14.7% vs. 4.3%, *p* < 0.005). Another retrospective study involving T2D with MAFLD showed an increased risk of heart block in individuals with MAFLD, compared with those without MAFLD (adjusted OR 3.04, 95% CI 1.81–5.10) [124].

**Figure 3:**
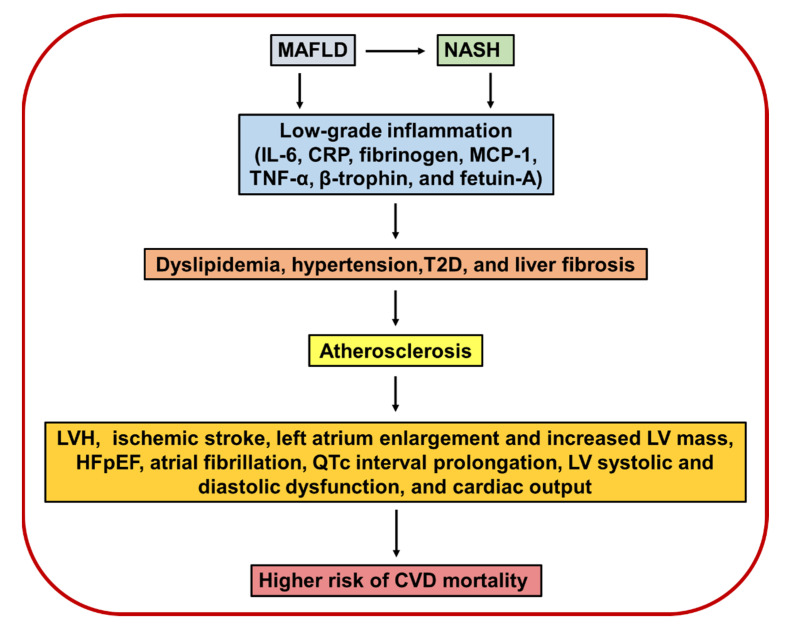
Cardiovascular adverse events associated with MAFLD and NASH. Systemic low-grade inflammation induced in MAFLD or NASH is linked with dyslipidemia, hypertension, T2D, and hepatic fibrosis, which may cause atherosclerosis and finally cardiovascular complications with higher risk of CDV mortality. Abbreviations: MAFLD, metabolic dysfunction-associated fatty liver disease; NASH, nonalcoholic steatohepatitis; IL-6, Interleukin-6; CRP, C-reactive protein; MCP-1, monocyte chemotactic protein 1; TNF-α, tumor necrosis factor-α; T2D, type 2 diabetes; LVH, left ventricular hypertrophy; LV, left ventricular; HFpEF, HF with preserved ejection fraction; QTc, corrected QT interval; CVD, cardiovascular disease.

Heart rate QTc prolongation has been established as a potent risk factor for ventricular arrhythmias and sudden cardiac death, and the severity of MAFLD was associated with an increased risk of QTc interval prolongation both in individuals with T2D and non-diabetic populations, which predisposed to an increased risk of sudden cardiac death [16,116]. Several studies have shown LV diastolic dysfunction and mild alterations in LV structure in patients with MAFLD, and without hypertension, diabetes, and morbid obesity [71]. A meta-analysis of 12 cross-sectional studies (involving 280,645 subjects) showed that there was a significant association between MAFLD and a higher risk of LV diastolic dysfunction [116]. Another study showed diastolic dysfunction in 48 never-treated hypertensive patients with MAFLD diagnosed by ultrasound, who also had LVH [133]. In addition, MAFLD is independently associated with LV systolic and diastolic dysfunction, with an increased cardiac output and myocardial remodeling [16]. Adverse structural remodeling of the heart is considered as a pivotal process in the development and progression of HF. In fact, patients with HF and coexisting MAFLD have a higher long-term risk of CVD outcomes, compared to their counterparts without coexisting liver disease [116]. Graner and colleagues reported that non-diabetic subjects with MAFLD have higher amounts of EAT as well as diastolic dysfunction, and liver triglyceride and VAT were independent predictors of LV diastolic function, whereas myocardial triglyceride was not associated with diastolic function [151]. In a Dutch population without known chronic liver diseases, hepatic triglyceride content was independently associated with LV diastolic dysfunction, mainly in subjects who were obese [16]. Meanwhile, in a cohort of approximately 21,000 South Korean adults, the severity of MAFLD was associated with abnormal LV relaxation and remodeling, independent of multiple cardio-metabolic risk factors [16]. Another cross-sectional study from southern Italy concluded that the severity of MAFLD correlated with EAT thickness, which was an indicator for diastolic dysfunction [133]. In line, several studies suggest that the MAFLD-related risk of LV diastolic dysfunction increased progressively with the severity of hepatic fibrosis [116]. One prospective Turkish study showed an association with NASH and subclinical systolic and diastolic LV dysfunction [125]. One of the consequences of diastolic dysfunction is AF [133]; as already mentioned, MAFLD has an increased prevalence of AF, mainly in T2D patients [14,57,124]. Additionally, some evidence suggests the association between MAFLD and certain types of cardiac conduction defects, mainly persistent first-degree atrio-ventricular block, left anterior hemiblock, or right bundle branch block, which are risk factors for cardiac mortality [116]. With respect to this, a meta-analysis of three cross-sectional studies (considering 3651 participants) reported that the risk of having the aforementioned cardiac conduction defects was higher in subjects with MAFLD than in those without MAFLD [139]. In summary, several studies demonstrate an association of MAFLD and NASH, especially in severe stages with CVD (Figure 3).

## 6. Insulin Hormone

Insulin is a potent anabolic hormone that after ingestion is synthesized in pancreatic β-cells of the Langerhans islets as pre-proinsulin (a single chain); subsequently, its signal peptide is removed in the endoplasmic reticulum to generate proinsulin, which has three domains (amino-terminal B chain, a carboxy-terminal A chain, and a connecting peptide (CP) in the middle). Within the endoplasmic reticulum, proinsulin is processed by specific endopeptidases that cleave the CP, thus generating the mature form of insulin [19,22]. Insulin induces its actions by binding to the insulin receptor, which activates receptor autophosphorylation and subsequently triggers a downstream signaling cascade through the phosphorylation of tyrosine residues of the IRS-1 or IRS-2, followed by phosphorylation of phosphatidylinositol 3-kinase (PI3K), Akt1 and Akt2, PKC, and the mammalian target of rapamycin (mTOR), including ribosomal protein S6 kinase beta 1 (S6K1). This signaling results in the translocation of the GLUT4 to the membrane, thus promoting glucose uptake in target tissues such as skeletal muscle, the myocardium, and adipose tissue. Insulin promotes glycogen synthesis in skeletal muscles, suppresses hepatic glucose production, and inhibits lipolysis in adipocytes [22].

### The Role of Insulin Resistance in MAFLD/NASH and in CVD

In insulin resistance conditions, some alterations in the genes associated with insulin signaling have been found. Insulin resistance is commonly related with increased adipose tissue lipolysis and FFA release, the formation of sdLDL, low levels HDL-cholesterol, decreased lipoprotein lipase activity (a major mediator of VLDL clearance), and a low-grade chronic inflammatory state due to the expression of pro-inflammatory cytokines, such as TNF-a and IL-1β, which is observed in obese, in diabetic, and in MAFLD subjects [19,22,152]. The lipid infusion increases in diacylglycerol levels and PKC signaling, leading to a defective activation of the IRS-1/Akt pathway in the skeletal muscle, have been reported in animal models and in humans [19].

Insulin resistance increases hyperglycemia, which triggers oxidative stress and causes an inflammatory response that leads to cell damage [22]. Moreover, hyperglycemia may influence coronary artery disease via the promotion of monocyte/macrophage adhesion to the endothelium, enhancement of vascular smooth muscle cell proliferation, and induction of endothelial dysfunction [18]. The endothelial dysfunction is related with CVD, including hypertension, atherosclerosis, and coronary artery disease, which are caused by insulin resistance [22]. Furthermore, insulin resistance in endothelial cells increases pro-thrombotic factors, pro-inflammatory markers, and ROS, which increase the intracellular levels of ICAM-1 and VCAM-1 [22]. In fact, the prevalence of insulin resistance is high in MAFLD and even higher in patients with NASH compared to those with simple steatosis [19]. Consequently, MAFLD/NASH may increase the risk of CVD and other cardiac complications by exacerbating hepatic and systemic insulin resistance, promoting atherogenic dyslipidemia, and releasing several pro-atherogenic, pro-coagulant, and pro-inflammatory mediators [116]. A homeostasis model assessment of insulin resistance (HOMA-IR) is a method to evaluate insulin resistance, and it was reported that the value of the HOMA-IR was significantly higher in NASH subjects than in healthy controls (4.4 ± 2.5 vs. 1.7 ± 0.6; *p* < 0.001), but this study could not show a significant correlation of HOMA-IR to the severity of either histologic grading or staging due to a small sample size (41 cases of biopsy-proven MAFLD). However, Kessoku et al. evaluated 1365 biopsy-proven MAFLD patients registered, and they demonstrated that HOMA-IR significantly increased depending on the degree of hepatic fibrosis. A study in Italy investigated 118 consecutive biopsy-proven MAFLD patients (25% with T2D), and it reported that HOMA-IR independently predicted advanced hepatic fibrosis. In addition, 361 biopsy-proven Japanese MAFLD patients without T2D were recently investigated, and the results showed that HOMA-IR ≥2.90 was an independent predictor of advanced fibrosis in nondiabetic MAFLD patients, and the authors suggest that there may be a pathway for insulin resistance to activate HSCs directly [20]. In line, in patients undergoing biliopancreatic diversion in a 5-year follow-up study, it was reported that remission of NASH is driven by the reversal of whole-body insulin resistance post-intervention [153]. Additionally, a meta-analysis of 65 studies, which included 516,325 participants, showed that insulin resistance, evaluated by the HOMA index, was a good predictor for CVD [22]. Therefore, these studies suggest that insulin resistance could be the main link between MAFLD/NASH and CVD. Interestingly, insulin resistance by itself is a major cardiovascular risk factor in healthy individuals and patients with diabetes [154]. With respect to this, a study in 1996 showed a direct relation between insulin resistance and atherosclerosis [22]. The risk of coronary artery disease is approximately three times greater in individuals with insulin resistance than in those who do not have insulin resistance. Insulin resistance was the most important single risk factor for coronary artery disease in young adults, being responsible for around 42% of myocardial infarctions [154]. In summary, a large number of studies support the notion that CVD is linked to insulin resistance; however, there are some controversial reports as well [22].

## 7. Strategies to Treat MAFLD and NASH and Cardiovascular Prevention

Currently, diverse promising MAFLD/NASH therapies with CVD-protecting properties are in development.

### 7.1. Lifestyle Modifications

Current therapies to counteract both the progression and development of MAFLD and CVD include lifestyle interventions, smoking cessation, weight loss, diet modifications, and physical exercise that remain the optimal therapeutic strategies according to clinical practice guidelines [100].

### 7.2. Smoking Cessation

Smoking contributes to the development of MAFLD, and it is a cardiovascular risk factor; thus, smoking cessation is important to the prevention of the primary causes of MAFLD and CVD [100,109].

### 7.3. Weight Loss

In humans, it is difficult to achieve and maintain weight loss in many cases. Energy restriction causes weight loss and the reduction of hepatic fat, independent of the diet; for instance, weight loss around 7–10% is desirable in overweight and obese patients with MAFLD to achieve a significant reduction in hepatic steatosis, thus delaying vascular and metabolic complications in MAFLD [100,155].

### 7.4. Diet Modifications

Guidelines for both the treatment of MAFLD and cardiovascular disease prevention currently recommend a low-carbohydrate, ketogenic, low-fat, high-protein, and Mediterranean diet, which induces a reduction effect on dyslipidemia, hepatic steatosis, and related comorbidities. Avoiding fructose-containing beverages and foods has been associated with a lower prevalence of MAFLD and helps to reduce CV risk [100,109,156].

### 7.5. Exercise and Physical Activity

The optimal therapeutic strategy recommended for MAFLD patients according to clinical practice guidelines includes a reduction in caloric intake in combination with exercise such as high-intensity interval, aerobic, and resistance training, which have been shown to improve plasma lipid status and insulin resistance. In addition, exercise improves the CVD risk factors, such as plasma levels of triglycerides-rich VLDL1 particles and LDL-cholesterol, and reduced arterial stiffness [109,157]. Additionally, exercise and physical activity are recommended to promote metabolic and vascular health, including benefits for NASH and fibrosis [100].

### 7.6. Medical Therapy

Current, pharmacological therapies for MAFLD/NASH treatment are conducted to reduce liver fat accumulation, stimulate metabolic pathways, and ameliorate liver injury.

#### 7.6.1. Aspirin

Acetylsalicylic acid (aspirin) is recommended once atherosclerotic disease is established, and it could help to reduce liver fibrosis. Therefore, the intake of acetylsalicylic acid might prevent CVEs [100].

#### 7.6.2. Statins

Statins are specific inhibitors of 3-hydroxy-3-methylglutaryl coenzyme A reductase (HMGCoA reductase), which is rate-limiting enzyme in cholesterol biosynthesis. They also promote the synthesis of the LDL-receptor, and they increase the expression of the membrane LDL-receptor, thus lowering cholesterol levels. Statins can decrease hepatic triglycerides, and they also have pleiotropic properties that account for their anti-inflammatory, anti-proliferative, anti-thrombotic, anti-oxidative, anti-cancer, and immuno-modulatory actions in vitro and in vivo. Therefore, they are essential components in the drug modification of cardiovascular risk, and they are recommended for patients with MAFLD and NASH [100,155]. In both the GREACE study and the IDEAL study, atorvastatin reduced cardiovascular risk in subjects with elevated transaminases. Therefore, statin therapy is recommended in patients with MAFLD due to both cardiological and hepatological benefits [100].

In addition, bempedoic acid, an inhibitor of ATP citrate lyase (enzyme involved in cholesterol synthesis upstream of HMGCoA reductase), reduces LDL-cholesterol consistently as a monotherapy and combined with statins and ezetimibe. In the CLEAR Tranquility study of statin-intolerant patients treated with ezetimibe, bempedoic acid decreased LDL-cholesterol by 28% without adverse effects [158].

#### 7.6.3. Ezetimibe

The use of ezetimibe, another lipid-lowering agent supported by guidelines, is safe and effective for the prevention of CVEs in MAFLD. Ezetimibe selectively inhibits cholesterol absorption from the intestine by binding to the brush border, thus lowering LDL-cholesterol. It also can increase lipoprotein assembly in the liver and perhaps in the intestine. Ezetimibe has also been shown to decrease hepatic lipid synthesis and inhibits the development of MAFLD in animal models. Studies in adult patients with MAFLD indicate an improvement in liver histology, but with a worsening or no effects on insulin sensitivity [100,155].

#### 7.6.4. PCSK9 Inhibitor

PCSK9 inhibitors represent a relatively new substance class in LDL-cholesterol-lowering therapy. Several studies suggest that high intrahepatic or circulating levels of PCSK9 influence muscle and liver lipid storage and may contribute significantly to the pathogenesis and progression of MAFLD. In large PCSK9 Fourier and Odyssey outcome studies no liver-related signals were observed; thus, it appears to be safe in the population with liver diseases [100]. Of note, one main caveat which limits the use of PCSK9 inhibitors is their cost, although it is decreasing [158].

Several pharmacologic approaches targeting PCSK9 are being evaluated. One is called inclisiran (a small-interfering RNA), which selectively targets hepatic mRNA encoding PCSK9. In the ORION-1 trial, inclisiran resulted in LDL-cholesterol reductions, and a phase III trial for assessing the effect of inclisiran on cardiovascular outcomes, ORION-4, is at the planning stage. The inclisiran is an initial repeat dose after 3 months and then administered only every 6 months. Another approach that implicates PCSK9 is the active vaccination. The vaccine AT04A, which is directed against PCSK9, has been proven successful in mice. Results of a phase I trial in humans were presented, although LDL-cholesterol was decreased by only 9% after 90 weeks; however, the principle is promising [158].

#### 7.6.5. Fibroblast Growth Factor 21 Analogues

Fibroblast growth factor 21 (FGF21) is an endocrine hormone of the FGF family, which is secreted mainly by the liver, and it plays an important role in glucose and lipid metabolism and insulin sensitivity. FGF21 analogues, one of the most promising drugs for MAFLD/NASH treatment with a documented effect on lowering CVD risk. A long-acting FGF21 analogue, PF-05231023, was tested on obese patients with and without T2D. PF-05231023 significantly decreased triglycerides levels and increased HDL-cholesterol and adiponectin. In addition, another PEGylated FGF21 analogue, pegbelfermin, was tested in a phase 2a study on MAFLD patients with obesity, which resulted in a significant reduction in liver fat. Meanwhile, AKR-001 (FGF21 analogue) had a positive influence on the lipoprotein profile (triglycerides, nHDL-cholesterol, HDL-cholesterol, ApoB, and ApoC3) and improved insulin sensitivity. Additionally, the anti-atherosclerotic effect of FGF21 was studied in several clinical trials, and it was shown to improve the cardio-metabolic profile in obese patients with T2D significantly, as reviewed in [109].

#### 7.6.6. Farnesoid X Receptor Agonists

The farnesoid X receptor (FXR) is a bile-acid-activated nuclear receptor, and, when bile acid binds to the FXR, it improves insulin sensitivity and reduces hepatic gluconeogenesis and circulating triglycerides by inhibiting the ChREBP and SREBP-1 genes’ expression, respectively [109,159]. These effects are mediated by decreased liver lipid synthesis and augmented peripheral clearance of VLDL. The beneficial effects of FXR activation by 6-ethyl-chenodeoxycholic acid (obeticholic acid: OCA), a potent activator of FXR, have been reported in rats with diabetes mellitus, obesity, insulin resistance, and liver steatosis. In addition, preliminary data on adults with MAFLD treated with OCA have shown promising results, but the long-term benefits and safety need clarification [109]. In addition, in a 6-week hyperinsulinemic, euglycemic clamp study of 64 subjects with MAFLD and diabetes, the OCA was associated with improved insulin sensitivity. A multicenter, randomized, double-blind, placebo-controlled phase IIb study, in which 283 patients received either 25 mg of OCA or a placebo for 72 weeks, was conducted. OCA was associated with a significant improvement in the NAFLD Activity Score (NAS), and there was a significant improvement in fibrosis stage. Currently, a phase III trial (REGENERATE) investigates the potential utility of OCA in patients with NASH, using a lower dose to determine whether it retains its efficacy with higher tolerability (NCT02548351). Another FXR agonist such as Tropifexor (LJN452), which is highly specific for FXR target genes in the liver and intestine, in small doses and with fewer systemic effects, is currently in phase II trials for NASH (NCT02855164) [159]. Therefore, FXR may offer new perspectives for the treatment of MAFLD.

#### 7.6.7. GLP1 Agonist

The glucagon-like peptide 1 (GLP1) is an incretin hormone secreted by intestinal L-cells at the post-prandial phase, which improves hyperglycemia by stimulating glucose-dependent insulin secretion and inhibiting glucagon secretion. Additionally, GLP1 stimulates weight loss by decreasing the gastric-emptying time while enhancing satiety by activation of GLP1 receptors in the hypothalamus [18]. The efficacy of GLP1 receptor agonists (GLP1-RA) in reducing serum liver enzymes and improving hepatic steatosis has been shown in several studies [18]. The GLP1 pharmacological agonist, liraglutide, was used via a subcutaneous injection daily for 24 weeks in glucose-intolerant NASH patients and significantly improved liver function and histological features. Similar results were reported for the GLP1-RA semaglitude, which decreased circulatory levels of ALT and hs-CRP in subjects at risk for MAFLD. The effectiveness of liraglutide and semaglutide is currently being examined in phase 2 clinical trials (NCT01237119 and NCT03987451) [160]. Currently, combinatorial therapy approaches are being tried to explore the role of combinations of other drugs with semaglutide in promoting improvements in fibrosis [18]. GLP1-RAs are associated with a significant reduction in CV risk. In fact, several trials have demonstrated a beneficial effect on CV outcomes using different GLP1-RAs such as liraglutide, semaglutide, liraglutide, and albiglutide. These beneficial effects of the GLP1-RAs on the reduction in CV outcomes were also confirmed by two meta-analyses [18].

#### 7.6.8. Bariatric Surgery

If a lifestyle intervention and adequate treatment of comorbidities are insufficient to decrease the overall risk, bariatric surgery in MAFLD is a final option. Bariatric surgery is effective for MAFLD and NASH treatment, especially when accompanied with severe obesity. Bariatric surgery also decreases the risk of CVD events among T2D and obese patients. Therefore, a similar effect would be also expected for NASH patients to reduce CV outcomes [100,109].

In summary, several pharmacological MAFLD/NASH therapies with CVD-protection are under development. However, the most efforts should be oriented on the promotion of a healthy lifestyle, nutrition literacy, and smoking cessation, thus contributing to the prevention of the primary causes of MAFLD/NASH and CVD.

## 8. Conclusions

Obesity is a chronic disease which includes many different anatomical, physiological, and pathological phenotypes; its pathophysiology is a complex interaction of genetics and obesogenic factors, including the dysregulation of adipokines and hormones as well as neuroendocrine signaling. As the prevalence of obesity increases worldwide, so too do comorbidities that affect quality of life. MAFLD and NASH are closely linked with metabolic disorders, such as obesity, dyslipidemia, hypertension, and diabetes. Severe obesity is associated with hemodynamic alterations, producing changes in cardiac morphology and ventricular function. This review supports the existence of an association between the presence and severity of MAFLD, particularly NASH and fibrosis, with an elevated risk of CVD. However, this association depends on the coexistence of a metabolic syndrome or T2D. Importantly, MAFLD independently increases the risk of atherosclerosis, cardiomyopathy, arrhythmia, which may result in cardiovascular morbidity and mortality, although the pathophysiological molecular mechanisms to elucidate the relationship between MAFLD and CVD are not clear yet. Since insulin resistance is common in patients with MAFLD or NASH and also is considered as a good predictor for CVD, the presence of insulin resistance may be the main underlying mechanism between MAFLD/NASH and CVD development. Furthermore, insulin resistance induces atherogenic dyslipidemia, pro-inflammatory markers, vasoactive factors and thrombogenic molecules, and ROS, which are involved in the CVD development. Thus, further research is needed in order to discover the exact pathophysiological molecular mechanisms behind this association to prevent CVD complications.

Considering the close link between cardio-metabolic disorders with MAFLD and obesity, lifestyle interventions to induce weight loss through diet and exercise are currently the gold standard method of treating these conditions. Therefore, further research, improvements, and clinical trials are required to develop optimal combination therapies for the treatment of human metabolic diseases linked to obesity.

## Figures and Tables

**Table 1:** Factors associated with the obesity development.

Types of Risk Factors	Specific Risk Factors
Genetics	Melanocortin-4 receptor mutation, leptin deficiency, pro-opiomelanocortin deficiency, variant rs9939609 of the FTO gene, parental obesity, and epigenetic modifications
Behavioral history	Nutrition, eating behavior, poor dietary choices, high calories, high-fat food, sugar-sweetened beverages, physical inactivity, sedentary lifestyle, insufficient sleep, stress, and smoking cessation
Socioeconomic	Low incomes, poverty, low education, unemployment, industrialization, mechanized transportation, urbanization, and socioeconomic status
Environmental	Cultural influences, television watching, fast food restaurants, culture, social bias, and environmental chemicals
Biological	Gut microbiome, viruses, brain-gut axis, prenatal determinants, pregnancy, gestational diabetes, menopause, neuroendocrine conditions, medications, and physical disability

Abbreviations: FTO, fat mass and obesity-associated.

**Table 2:** Comorbidities related to obesity.

Type of Comorbidity	Specific Comorbidity
Endocrine	Hyperleptinemia, hypothyroidism, hypercortisolism, Cushing’s syndrome, polycystic ovary syndrome, metabolic syndrome, and T2D
Gastrointestinal	Kidney stones, glomerulopathy, kidney dysfunction, urinary incontinence (in women), pancreatitis, gallbladder disease, and liver disease (MAFLD and NASH)
Respiratory	Obstructive sleep apnea and asthma
Esophageal	Gastroesophageal reflux disease and Barrett’s esophagus
Cardiovascular	Hypertension, CHD, AF, diastolic dysfunction, HF, ischemic stroke, and cardiac fibrosis
Neurological	Alzheimer’s disease, vascular dementia, any type of dementia, mood, anxiety, and other psychiatric disorders
Fertility	In women: preeclampsia, eclampsia of pregnancy, depression, amenorrhea, menorrhagia, and infertility In men: low sperm count and erectile dysfunction
Immune system dysregulation	Infections such as surgical-site, urinary tract, nosocomial, and skin
Autoimmune diseases	Rheumatoid arthritis, osteoarthritis, multiple sclerosis, psoriasis, and psoriatic arthritis
Viral	H1N1 influenza virus and SARS-CoV-2 (obesity increases severity of the disease)
Cancer	Esophageal, colon, pancreatic, endometrium, renal, gastric, uterine, gallbladder, cervical, thyroid, prostate, leukemia, liver, ovarian (epithelial), and breast (postmenopausal)

Abbreviations: T2D, type 2 diabetes; MAFLD, metabolic dysfunction-associated fatty liver disease; NASH, nonalcoholic steatohepatitis; CHD, coronary heart disease; AF, atrial fibrillation; HF, heart failure; H1N1, influenza A; SARS-CoV-2, severe acute respiratory syndrome coronavirus 2.

## Data Availability

Not applicable.
